# Oral Dysplastic Complications after HSCT: Single Case Series of Multidisciplinary Evaluation of 80 Patients

**DOI:** 10.3390/life10100236

**Published:** 2020-10-09

**Authors:** Stefania Leuci, Noemi Coppola, Andrea Blasi, Elvira Ruoppo, Maria Eleonora Bizzoca, Lorenzo Lo Muzio, Luana Marano, Antonio Maria Risitano, Michele Davide Mignogna

**Affiliations:** 1 Department of Neurosciences, Reproductive and Odontostomatological Sciences, Oral Medicine Unit, Federico II University of Naples, 80138 Naples, Italy; stefania.leuci@unina.it (S.L.); andreablasi79@gmail.com (A.B.); elvira.ruoppo@gmail.com (E.R.); mignogna@unina.it (M.D.M.); 2 Department of Clinical and Experimental Medicine, University of Foggia, 71122 Foggia, Italy; marielebizzoca@gmail.com (M.E.B.); lorenzo.lomuzio@unifg.it (L.L.M.); 3 Department of Clinical Medicine and Surgery, Federico II University of Naples, 80138 Naples, Italy; luanamarano@libero.it; 4 Hematology and Bone Marrow Transplant Unit, Moscati Hospital, 83100 Avellino, Italy; amrisita@unina.it

**Keywords:** OSCC, GVHD, HSCT, oral cancer, head and neck cancer

## Abstract

Oral squamous cell carcinoma (OSCC) is the most common secondary solid malignancy after hematopoietic stem-cell transplantation (HSCT). OSCC following HSCT is frequently preceded by chronic graft-versus-host disease (cGVHD). The aim of this study was to describe a cohort of post-HSCT patients and to evaluate the onset of oral epithelial dysplasia and/or OSCC over time. In this retrospective cohort study, we present a cohort of hematological patients that underwent HSCT. Demographic variables, clinical hematological data, data regarding acute graft-versus-host disease (aGVHD) and cGVHD, and oral clinical features were analyzed. We focused on clinicopathological features of a subgroup of 22 patients with oral cGVHD and OSCC after HSCT. Among 80 included patients, 46 patients (57.5%) developed aGVHD and 39 patients (48.7%) developed cGVHD. Oral mucosa was involved in 17 patients with aGVHD (36.9%) and in 22 patients (56.4%) with cGVHD. Out of a total of 22 oral biopsies, roughly 40% revealed mild to moderate dysplasia, and 32% were OSCC. In the absence of international agreement on the best timing of oral follow-up after HSCT, it is mandatory to establish a close multidisciplinary evaluation in order to prevent the onset of HSCT-related OSCC and to reduce post-transplant mortality due to secondary tumors.

## 1. Introduction

Graft-versus-host disease (GVHD), similar to an autoimmune disorder, is the main complication and the leading cause of mortality after hematopoietic stem-cell transplantation (HSCT) [1]. In fact, human leukocyte antigen (HLA) disparity between donor and recipient cells induce a donor T-cell reaction against the host’s tissue antigens, recognized as a non-self, resulting in cell death via apoptosis. All these events generate multiple clinical, pathological, and immunological manifestations that characterize the immunophenotype of the disease [2].

GVHD can be distinguished as acute (aGVHD) and chronic (cGVHD) forms according to the time of onset of the disease and the signs and symptoms [2,3]. aGVHD, T-cell mediated, occurs typically before 100 days after HSCT in approximately 50–70% of transplant recipients, with mortality rates of 27–92%, and mainly affects the gastrointestinal (GI) tract, the skin, and the liver [4,5]. Its severity is divided into four grades (I-IV) according to the impairment and dysfunction of the involved organs; grade I is generally associated with a low risk of relapse, while grades II-IV are most related to post-transplant morbidity [1,6,7]. cGVHD, instead, is immunologically more complex (B and T-cell-mediated) and occurs 100 days after HSCT in more than half of recipients, with an aggregate cumulative incidence of 30% to 50% and a 5-year mortality rate of 70% [8]. cGVHD is characterized by multi-organ involvement and by signs and symptoms affecting the GI tract, skin, liver, lung, genitalia, muscles, and joints, and, above all, oral cavity [1]. 

Oral cGVHD manifests in 45–83% of transplant patients, and the most important clinical manifestations are represented by lichenoid oral mucositis, Sjögren-like signs, and scleroderma. In particular, with regard to lichenoid changes, they are frequently localized in the labial and buccal mucosa, the tongue, and the palate with different clinical aspects—atrophic, erythematous, erosive, ulcerative, keratotic reticular-, or plaque-type—with or without pain [9,10,11]. Furthermore, in patients with oral cGVHD, there is a greater incidence of pyogenic granuloma, verruciform xanthoma, and oral squamous cell carcinoma (OSCC) [5,12].

The long-term follow-up increases the likelihood of diagnosing secondary cancers in transplant recipients with a high risk of death. According to scientific data, about 2–6% of transplant recipients develop a secondary solid tumor 10 years after HSCT; among secondary solid malignancies, oropharyngeal cancer is the most frequent, and OSCC represents half of these [13]. Young patients with oral cGVHD with no gender predilection and no risk factors are at considerable risk for developing OSCC. The preferential sites of onset are the buccal mucosa, lips, and tongue, with clinical phenotypes resembling other forms of OSCC with exophytic or endophytic ulcerated lesions. It frequently arises on a mucosa site of previous dysplasia and is part of a field cancerization. In fact, the OSCC following HSCT is commonly multifocal or metachronous and has a high rate of recurrence [13,14,15]. All these features suggest that HSCT-related OSCC is more aggressive.

The aim of this study is to comprehensively present our institutional experience with a cohort of post-HSCT patients, reviewing the clinical features, GVHD onset, and type, and to evaluate data on a subset of patients affected by oral cGVHD, analyzing the onset of oral epithelial dysplasia and/or OSCC over time.

## 2. Materials and Methods 

A retrospective single-center cohort study was conducted at the Oral Medicine Unit, Department of Neuroscience, Reproductive, and Odontostomatological Sciences, in collaboration with the Hematopoietic Stem Cell Program at the Federico II University of Naples. It is compliant with the ethical principles of the World Medical Association Declaration of Helsinki and was approved by the Ethics Committee of the Federico II University of Naples (N°363/19). The reporting of data followed the guidelines of the STROBE statement.

We have analyzed the medical records of a cohort of consecutive patients that underwent HSCT from 2011 to 2017 because of different malignant and non-malignant hematological diseases, such as acute and chronic myeloid leukemia (AML, CML), multiple myeloma (MM), acute and chronic lymphoid leukemia (ALL, CLL), Hodgkin lymphoma and non-Hodgkin lymphoma (HL, NHL), acute myeloid leukemia after myelodysplasia (AML POST MDS), Fanconi anemia (FA), aplastic anemia (AA), myelofibrosis (MF), myelodysplasia (MDS), and dyskeratosis congenita (DC).

The following data were recorded:

1. Demographic variables: age, sex.
2. Clinical hematological data: underlying disease, type of conditioning regimen, stem cell source (bone marrow or peripheral blood), donor’s sex, HLA matching, post-transplant response, recurrence, mortality.
3. Data regarding aGVHD and cGVHD: drug prophylaxis, organs involvement, grading documented according to the 2014 National Institutes of Health consensus criteria, treatment protocol.

All the patients underwent a complete clinical oral examination, and, if they had oral lesions, an incisional or excisional biopsy with a histological examination and HPV detection was performed. The following oral data were described: morphology, site, histological features, symptoms (pain, anesthesia/paresthesia). In the subgroup of OSCC patients, data on the onset of the disease, the staging and management, the risk factor exposure (tobacco and alcohol), the time interval from HSCT to the onset of oral signs, and the oral medicine referral’s delay (time elapsed between the onset of oral lesions and the request for oral medicine advice) were recorded.

In the overall studied population, simple linear regressions were calculated to predict cGVHD, relapsem and death based on age, gender, pathological conditions, the type of donor, matching between the donor and receiver, the source of hematopoietic stem cells, the type of conditioning, conditioning regimen, drug prophylaxis, previous aGVHD and its treatment, and therapy for cGVHD and cGVHD-GS.

A further analysis was conducted on a cGVHD population calculating simple linear regressions using the same variables in addition to alcohol consumption, smoking habits, and histological diagnosis.

A statistical analysis was performed using the Statistical Package for Social Sciences Software (IBM Corp. Released 2012. IBM SPSS Statistics for Windows, Version 21.0. Armonk, NY, USA: IBM Corp); *p* < 0.05 was set as the level of statistical significance. 

## 3. Results

The demographic and clinical characteristics of the patients are shown in Table 1. A total of 80 patients undergoing HSCT with average age of 40.8 years (18.4–65.1) were included. Bone marrow and peripheral blood stem cells were the stem cell source in 65% and 35%, respectively, and myeloablative conditioning was used in the majority of patients (81.2%). The median follow-up for surviving patients was 22 months.

Forty-six patients (57.5%) developed aGVHD: 18 (39.1%) grade I, 20 (43.5%) grade II, and 8 (17.4%) grade III. Although the simultaneous involvement of multiple organs is possible for each patient, the skin was the most frequently affected site (84.9%) (Table 2). Thirty-nine patients (48.7%) developed cGVHD, not always preceded by aGvHD; the cGVHD grades were mild in 16 cases (41%), moderate in 15 cases (38.5%), and severe in 8 cases (20.5%). Oral mucosa was the principal target organ in 17 patients with aGVHD (36.9%) and in 22 patients (56.4%) with cGVHD (Table 2). 

Data regarding the oral cGVHD are shown in Table 3. In each patient, multiple oral lesions were found. The most frequent oral clinical manifestations were keratotic lesions (42.9%), followed by erosive/ulcerative lesions (21.4%), verrucous lesions (21.4%), atrophic lesions (7.1%), and bullous lesions (7.1%). On histological examination, 6 (27.3%) of the 22 biopsied lesions were non-dysplastic, 9 (40.9%) demonstrated mild to moderate dysplasia, and 7 (31.8%) were OSCC. In all the examined samples, no HPV was detected. 

Data regarding OSCC following HSCT are shown in Table 4. Conventional oral examination in patients with OSCC showed heterogeneous oral lesions with a predominant warty keratotic phenotype. None of the OSCC cases were preceded by an oral potentially malignant lesion. In the OSCC subgroup, five patients received myeloablative conditioning and two received non-myeloablative conditioning. Three patients received a sex-matched HSCT. Four were recipients of HLA-identical sibling transplants, in three patients the stem cell source was bone marrow, and in four patients it was peripheral blood. The average time elapsed between HSCT and the onset of oral lesions was 9.7 months; instead, as regards exclusively oral cancer, the median time from HSCT to the diagnosis of OSCC was 5 years. The average time to referral in oral medicine was 18 days.

In the overall sample population, a statistically significant regression (F(1, 75) = 11.722, *p* = 0.001) was found when predicting cGVHD based on aGVHD with an R2 of 0.124.

Additionally, when predicting death based on cGVHD a statistically significant regression (F(1, 75) = 10.911, *p* = 0.001) was found with an R2 of 0.115.

When analyzing the cGVHD population, statistically significant regressions (F(1, 20) = 1.274, *p* = 0.018) were found when predicting relapse based on mismatching between the donor and receiver genders, with an R2 of 0.250, and when predicting relapse based on cGVHD-GS (F(1, 20) = 7.682, *p* = 0.012), with an R2 of 0.278.

Finally, a statistically significant regression (F(1, 20) = 2.305, *p* = 0.001) was found when predicting death based on mismatching between the donor and receiver genders, with an R2 of 0.423.

## 4. Discussion

The increase in post-transplant survival has led to a greater number of patients with a well-recognized and potentially chronic severe complications, the most common of which is cGVHD. The prevalence of cGVHD in the included sample is 48.7%, in line with the published literature. Its occurrence increases with certain risk factors in both the donor and recipient; in particular, in the recipient, older age, the type of pre-transplant conditioning regimen, the use of radiotherapy, and the type of regimen prophylaxis are the major recognized risk factors. The donor-related risk factors are represented by gender difference between the donor and recipient, the number of previous pregnancies, the degree of compatibility of HLA, and the use of peripheral blood cells [16]. Although the data of our study confirm that a previous aGVHD is a predisposing factor for cGVHD, a reduced percentage of cases (12.5%) occurs ex novo without preceding aGVHD, as highlighted also by Lee S. [17].

cGVHD is a multi-organ manifestation, however the oral cavity is the most commonly involved site and 56.4% of the included patients with cGVHD developed oral lesions. Clinically, damage to the oral mucosa is manifested by a wide spectrum of lesions with or without symptoms, and lichenoid/hyperkeratotic plaques are the most common features. Oral cGVHD is characterized by multiple manifestations in each patient with polymorphic clinical aspects, usually treated with systemic and topical immunosuppressive/immunomodulatory therapies. 

In our cohort, the systematic assessment of oral pathology revealed oral malignancies in 9% of patients: oral examination showed exophytic lesions (*n* = 3) and well-defined plaque with small ulcerations (*n* = 4), and histological examination showed carcinoma in situ (CIS) (*n* = 4) and invasive OSCC (*n* = 3). In according to the TNM system, four cases were classified as stage 0, 1 case was stage I, and 2 cases were stage IVA. Surgical treatment was the elective therapy for all cases; neck dissection and radiotherapy were added only for stage IVA. All the patients are in clinical and instrumental quarterly follow-up, without showing signs of disease recurrence. In the cohort patients with oral cGVHD, the dysplastic transformation occurred only in patients with keratotic morphology. None of the patients with a erosive, atrophic, or bullous phenotype developed OSCC in the follow-up period. These data suggest that keratotic lesions could be a negative prognostic factor for OSCC onset during GVHD and, for this reason, it is mandatory to perform a careful clinical and histopathological evaluation. The average time from HSCT to onset of OSCC was 5 years; the median latency time between HSCT and the appearance of oral malignancy is in line with the data reported in previous studies [14,18]. The median time to referral in oral medicine was 18 days. Risk factors such as smoking, alcohol, and HPV are absent in all our OSCC patients, unlike the paper from Mawardi H et al., where a high prevalence of smoking and alcohol consumption were reported [13]. The absence of risk factors in our cohort is in line with the study by Chaulagain et al., and it makes prevention more difficult because acting on conventional risk factors may not be effective [14]. These data suggest that while the role of tobacco, alcohol, and viral infection in not HSCT-related head and neck cancer is well coded, their role in the HSCT population is controversial. Therefore, the mechanism of oral carcinogenesis post-HSCT may not be promoted by conventional risk factors, and other factors that could have a role have been discussed in the literature [18], such as the past history of cGVHD, radiotherapy, previous long-term immunosuppressive therapy ≥ 24 months, advanced age at transplant, genetic susceptibility, and time after HSCT [19]. All our OSCC cases had a previous multiorgan cGVHD with cutaneous, hepatic, ocular, gastrointestinal and pulmonary involvement and all cases had a previous immunosuppressive therapy. This finding confirms that oral cGVHD has an important role in HSCT-related carcinogenesis, probably due to local immunologic dysregulation [20]. cGVHD causes a long-term inflammation of the oral mucosa, with an upregulation of cytokines and the generation of reactive oxygen species, which are considered risk factors for the development of dysplastic lesions [21]. Furthermore, recent studies have confirmed the role of genomic instability in the development of OSCC following HSCT and the contribution of GVHD to this process [22]. In fact, the immunologically mediated chronic injury during cGVHD can induce a set of somatic alterations within the genome and predisposition to oncological transformation [23]. Moreover, the biological pathways of oral cGVHD are very unique—in fact, the FGCR2A genotype was found to be related only to oral cGVHD and not to other sites [24]. These data support the hypothesis that different biological mechanisms are responsible for the development of cGVHD and are associated with a different risk of cancer onset [24]. In our sample, 86% of oral cGVHD patients were treated for oral lesions with topical therapy; this suggests that oral manifestations have been remarkable and, therefore, the degree of inflammation was high. Rare cases of OSCC not preceded by oral cGVHD are described by Reddy et al. [25]. Regarding chronic immunosuppressive therapy, it is difficult to affirm a clear correlation between specific immunosuppressive systemic molecules and the cancer development for several reasons. Firstly, patients underwent multiple immunosuppressive regimens in single and multiple associations, therefore it is not possible to correlate the mechanism of action of each molecule with the onset of the oral disease. Furthermore, the pharmacological protocols used before and after transplantation are variable, and the effect also depends on the susceptibility of the recipient. In consideration of the heterogeneity of the data, it was not possible to establish an ideal prophylaxis to use to prevent the onset of oral dysplastic lesions. Compared to the current evidence, in our sample no association has been found between HSCT-related OSCC and the remaining risk factors. The mechanism underlying carcinogenesis in the HSCT population is still poorly understood and, in addition to the risk factors described above, the donor cells present in the recipient play a crucial role in systemic and oral carcinogenesis [26]. In this regard, it is interesting to note that, in our study, four of the seven patients who developed OSCC were the recipients of HLA-identical sibling transplants. It is known that the degree of HLA disparity is the key determinant for GVHD risk and, in second line, for the onset of cancer [27]; therefore, in our cases, the development of OSCC in patients with a full HLA match between donor and recipient is attributable to a pathogenetic mechanism not associated with donor cells. 

To prevent the onset of OSCC following HSCT and to reduce the post-transplant mortality due to secondary tumors, it is necessary that patients must undergo a regular and close follow-up, looking at modifications in clinical morphology, evaluating inhomogeneous patterns, and treating suspicious lesions with incisional biopsy in order to rule out the presence of dysplasia and local malignancies. There are still no agreed opinions on the timing of follow up after HSCT; however, pending an international consensus, it is necessary to follow these patients with a close multidisciplinary follow up, including hematologists, dermatologists, gastroenterologists, ENT, ophthalmologists, and oral medicine specialists.

The major limitation of this study is the heterogeneity of the included sample, evaluated in a retrospective way; after all, it is the only way to examine hematological patients with a high-mortality disease also with an extremely variable course. 

In the future, it will be interesting to explore the genotype of patients to investigate the molecular pathways underlying the onset of HSCT-related OSCC, trying to identify possible actions which may mitigate the risk associated with the transplant procedure.

## Figures and Tables

**Table 1:** Demographic and disease characteristics of the enrolled patients.

	N	(%)
**All patients**	80	
**Age, median years (range)**	40.8 (18.4–65.1)	
**Recipient sex**		
M	41	(51.2)
F	39	(48.7)
**Sex match between recipient and donor**		
Matching	41	(51.2)
Mismatching	39	(48.7)
**Disease**		
AML	32	(40)
CML	9	(11.2)
ALL	6	(7.5)
ALL ph+	5	(6.2)
CLL	1	(1.2)
MM	4	(5)
AA	6	(7.5)
HL	3	(3.7)
NHL	2	(2.5)
DC	1	(1.2)
AML POST SMD	6	(7.5)
MF	1	(1.2)
MDS	1	(1.2)
FA	3	(3.7)
**Stem cell source**		
BM	52	(65)
PB	28	(35)
**HLA compatibility**		
HAPLO	30	(37.5)
MUD	19	(23.7)
HLA-id	31	(38.7)
**Type of conditioning regimen**		
NMC	12	(15)
MAC	65	(81.2)
RIC	3	(3.7)
**Conditioning regimen**		
Benda/Flu/Mel	2	(2.5)
Tbf	42	(52.5)
Bu/Cy	8	(10)
Bu/Flu	11	(13.7)
Cy	2	(2.5)
Flu/Cy	7	(8.7)
Flu/Cy/Tbi	3	(3.7)
Flu/Tep	5	(6.2)
**GVHD Prophylaxis**		
CsA	1	(1.2)
Alemtuzumab	4	(5)
Alemtuzumab+ATG	1	(1.2)
MMF+ATG	2	(2.5)
MMF+Cy	34	(42.5)
MTX	21	(26.2)
MTX+ATG	14	(17.5)
MTX+ATG+Alemtuzumab	1	(1.2)
MTX+Alemtuzumab	1	(1.2)
MTX+MMF+ATG	1	(1.2)
**Toxicity post-HSCT ^1^**		
Liver	4	(5)
Cardiac	4	(5)
GI	32	(40)
Mucosal	56	(70)
Kidney	9	(11.2)
Vascular	5	(6.2)
Hypertension	2	(2.5)
Lyell’s syndrome	1	(1.2)
**Outcome post-HSCT**		
Complete remission	60	(75)
Partial remission	4	(5)
Data not available	14	(17.5)
Graft Failure	1	(1.25)
Death	1	(1.25)
**Relapse**	15	(18.7)
**Death**	36	(45)
**Median follow up of survivors, months (range)**	22 (1–78)	

^1^ Each patient had multiple post-HSCT toxicities, so the percentage does not correspond to the whole sample. **AML:** acute myeloid leukemia; **CML:** chronic myeloid leukemia; **ALL:** acute lymphoid leukemia; **ALL ph+:** acute lymphoid leukemia with Philadelphia chromosome-positive; **CLL:** chronic lymphoid leukemia; **MM:** multiple myeloma; **A:** aplastic anemia; **HL:** Hodgkin lymphoma; **NHL:** non-Hodgkin lymphoma; **DC:** dyskeratosis congenita; **AML POST SMD:** acute myeloid leukemia after myelodysplasia; **MDS:** myelodysplasia; **FA:** Fanconi anemia; **NMC:** nonmyeloablative conditioning; **MAC:** myeloablative conditioning; **RIC:** reduced intensity conditioning; **Benda:** Bendamustine; **Flu:** Fludarabine; **Mel:** Melphalan; **Tbf:** Thiotepa-Busulphan-Fludarabine; **Bu:** Busulphan; **Cy:** Cyclophosphamide; **Tbi:** Total body irradiation; **Tep:** Thiotepa; **HAPLO:** Haploidentical donor; **MUD:** Matched unrelated donor; **HLA-id:** HLA identical; **BM:** Bone marrow; **PB:** Peripheral blood; **CsA:** Cyclosporine; **ATG:** Anti-thymocyte globulin; **HSCT:** hematopoietic stem-cell transplantation; **MMF:** Mycophenolate mofetil; **Mtx:** Methotrexate; **GI:** gastrointestinal tract.

**Table 2:** Clinical features of aGVHD and cGVHD.

	N	(%)
**aGVHD**		
**All patients**	46	(57.5)
**Age, median years (range)**	41.9 (18.4–61.8)	
**Sites involved ^1^**		
Skin	39	(84.9)
Liver	19	(41.3)
Gastrointestinal tract	17	(36.9)
**Grading**		
I	18	(39.1)
II	20	(43.5)
III	8	(17.4)
IV	0	
**Therapy**		
First therapy	43	(93.5)
6-MP	41	(89.1)
Alemtuzumab	2	(4.3)
Second therapy	5	(10.9)
Begelomab	2	(4.3)
Alemtuzumab	1	(2.2)
MMF	1	(2.2)
ATG	1	(2.2)
Third therapy	1	(2.2)
MMF	1	(2.2)
**cGVHD**		
**All patients**	39	(48.7)
**Age, median years (range)**	41.4 (23.2–65.1)	
**Sites involved**		
Skin	31	(81.6)
Liver	27	(71.1)
Oral cavity	22	(56.4)
Eyes	15	(39.5)
Gastrointestinal tract	9	(23.7)
Lungs	7	(18.4)
Genitalia	6	(15.8)
Muscles, fascia, joints	2	(5.3)
**Grading**		
Mild	16	(41)
Moderate	15	(38.5)
Severe	8	(20.5)
**Therapy ^2^**		
Immunosuppressors	39	(100)
6-MP	33	(84.6)
CsA	37	(94.8)
MMF	20	(51.3)
RAP	1	(2.6)
mAB/pABs	9	(23.1)
Begedina	1	(11.1)
ATG	4	(44.4)
Rituximab	3	(33.3)
Alemtuzumab	1	(11.1)
Chemoterapies	15	(38.4)
Mtx	5	(33.3)
Cy	10	(66.6)
Steroids	21	(53.8)
TKI	5	(12.8)
ECP	8	(20.5)
PUVA	4	(10.2)
IVIG	1	(2.5)
DLI	1	(2.5)

^1^ The involvement of several organs at the same time is possible for each patient, so the numbers and percentages reported cannot be assimilated to the whole sample. ^2^ Each patient was treated with multiple drug treatments, so the numbers and percentages obtained are not in reference to the whole sample. **6-MP:** 6-mercaptopurine; **MMF:** Mycophenolate mofetil; **ATG:** Anti-thymocyte globulin; **CsA:** Cyclosporine; **RAP:** Rapamycin; **mABs:** monoclonal antibodies; **pABs:** polyclonal antibodies; **Mtx:** Methotrexate; **Cy:** Cyclophosphamide; **TKI:** tyrosine kinase inhibitor; **ECP:** extracorporeal photopheresis; **PUVA:** psoralen-UVA photochemotherapy; **IVIG:** IntraVenous ImmunoGlobulin; **DLI:** Donor Lymphocyte Infusion.

**Table 3:** Demographic, clinical, and histopathological features of patients with oral cGVHD.

	N	(%)
**All patients**	22	
**Age, Median years (range)**	41.3 (24–65.1)	
**Time lapse between HSCT and oral involvement (months)**	9.7 (3.3–36)	
**Average time to request advice in oral medicine (days)**	18.5 (1–90)	
**Morphology of oral lesions ^1^**		
Keratotic reticular lesions	7	(25)
Keratotic plaque lesions	5	(17.9)
Erosive/Ulcerative lesions	6	(21.4)
Verrucous lesions	6	(21.4)
Atrophic lesions	2	(7.1)
Bullous lesions	2	(7.1)
**Sites of oral lesions ^1^**		
Check	14	(31.8)
Dorsal tongue	7	(15.9)
Ventral tongue	6	(13.6)
Floor of the mouth	2	(4.5)
Hard palate	4	(9.1)
Soft palate	3	(6.8)
Lip	2	(4.5)
Gingival mucosa	6	(13.6)
**Histopathological diagnosis**		
No dysplasia	6	(27.3)
Mild/moderate dysplasia	9	(40.9)
CIS	4	(18.2)
OSCC	3	(13.6)

^1^ The involvement of several intraoral sites at the same time is possible for each patient, so the numbers and percentages reported cannot be assimilated to the whole sample. **HSCT:** hematopoietic stem-cell transplantation; **CIS:** carcinoma in situ; **OSCC:** Oral squamous cell carcinoma.

**Table 4:** Case series of patients with HSCT-related OSCC.

Treatment	Surgical Excision	Resection, Neck Dissection and Radiotherapy	Resection, Neck Dissection and Radiotherapy	Surgical Excision	Surgical Excision	Surgical Excision	Surgical Excision
Staging (TNM)	0	IV A	IV A	I	0	0	0
Intraoral site	Check	Alveolar ridge	Check	Gingival mucosa	Ventral tongue	Ventral tongue	Lip
cGVHD	Oral cavity, skin, liver, eyes, GI, lungs	Oral cavity, skin, hepatic, eyes, GI	Oral cavity, skin, liver	Oral cavity	Oral cavity, skin, liver, eyes, genitalia, lungs	Oral cavity, skin, liver	Oral cavity, skin, GI
aGVHD	Liver	Liver, GI	-	-	Skin	-	-
HLA compatibility/sex of donor	HLA id/F	MUD/M	HLA id/M	HAPLO/M	HLA id/M	MUD/M	HLA id/M
Conditioning regimen	MAC	NMC	MAC	MAC	MAC	NMC	MAC
Latency of OSCC (year)	6	3	4	7	4	5	6
Age/sex	59/M	37/M	24/F	51/M	40/M	28/F	26/F
**Patients**	1	2	3	4	5	6	7

**OSCC:** Oral squamous cell carcinoma; **NMC:** nonmyeloablative conditioning; **MAC:** myeloablative conditioning; **HLA-id:** HLA identical; **MUD:** Matched unrelated donor; **HAPLO:** Haploidentical donor; **GI:** Gastro-intestinal tract.
